# Data on donation behavior towards the conservation of migratory species

**DOI:** 10.1016/j.dib.2023.109130

**Published:** 2023-04-07

**Authors:** Anna Lou Abatayo, Mathias Vogdrup-Schmidt, Jason F. Shogren, Niels Strange, Bo Jellesmark Thorsen

**Affiliations:** a Environmental Economics and Natural Resources Group, Wageningen University and Research, Hollandseweg 1, Wageningen 6706KN, Netherlands; b Department of Food and Resource Economics, University of Copenhagen, Rolighedsvej 23, 1958 Copenhagen, Denmark; c Center for Macroecology, Evolution and Climate, Universitetsparken 15, Bld. 3, 2nd floor, DK-2100 Copenhagen, Denmark; d Department of Economics, University of Wyoming, 1000 E. University Ave., Laramie, WY 82071, USA

**Keywords:** Economic experiments, Dictator game, Information, International collaboration

## Abstract

The data contains 716 individual decisions and responses from a lab-in-field experiment and an exit questionnaire that were conducted in Denmark, Spain, and Ghana. Individuals were initially asked to perform a small effort task (i.e., correctly counting the number of 1’s and 0’s in a page) to earn money and subsequently asked how much of their earnings they were willing to donate to BirdLife International to conserve Danish, Spanish, and Ghanaian habitats of the Montagu's Harrier, a migratory bird. The data is useful in understanding individual willingness-to-pay to conserve the habitats of the Montagu's Harrier along its flyway and could aid policymakers in having a clearer and more complete idea of support for international conservation. Among other things, the data can be used to look at the effect of individual socio-demographic characteristics and environmental and donation preferences on actual donation behavior.

**Specifications Table**

**Table:** 

Subject	Behavioral Finance and Economics, Microeconomics, Economics
Specific subject area	Individual donations to BirdLife International to conserve habitats of the Montagu's Harrier.
Type of data	Table (38 variables, 716 observations)
How the data were acquired	Data was acquired through lab-in-field experiments in Denmark, Spain, and Ghana. Two experiments were conducted: (1) a small effort task and (2) a dictator game. Copies of the consent form, experiment instructions, and exit questionnaire (in English) are available as supplementary materials of the original article [1]. Danish and Spanish translations are available upon request. Experiments in all three countries were run by hand. Each country had an instructor, who interacted with the participants, and an experimenter, who paid the participants and communicated with the experimenters in the other two countries. All instructors and experimenters were trained at the same time in Denmark.
Data format	Raw
Description of data collection	Participants were recruited from a database of experiment participants in Denmark and Spain, and through flyers and in-class announcements in Ghana. Participants were randomly assigned into experiment treatments. During the experiment, participants were not allowed to communicate with one another. Participants were only allowed to communicate with the instructor. Participants were only known by their subject ID number.
Data source location	The experiments were conducted in three countries:Denmark • Institution: University of Copenhagen • City: Frederiksberg Spain • Institution: Pompeu Fabra University • City: Barcelona Ghana • Institution: University of Ghana • City: Accra
Data accessibility	Repository Name: Mendeley Data Data identification number: 10.17632/xfhdfgps43.2 Direct URL to data: https://data.mendeley.com/datasets/xfhdfgps43/2[2]
Related research article	M. Vogdrup-Schmidt, A.L. Abatayo, N. Strange, J.F. Shogren, B.J. Thorsen, Factors affecting support for transnational conservation targeting migratory species. Ecological Economics. 157 (2019) 156-164. https://doi.org/10.1016/j.ecolecon.2018.11.011. [1]

## Value of the Data

• The data is useful to understand individual willingness-to-pay to conserve habitats of migratory species.
• The data is useful for academics, researchers, and policymakers who want to understand what factors affect support for international conservation as well as for policymakers and legislative practitioners working on designing international agreements on conservation along migratory flyways.
• The data is useful for international non-governmental organizations whose major funding sources come from individual donations.
• The data can provide insight into support for international collaboration for the conservation of endangered migratory species.
• The data can be used to understand which individual characteristics, donation preferences, and environmental preferences affect support for international conservation.
• The data can be combined with other data on small effort task and dictator game experiments around the world to make international comparisons and to understand an individual's willingness to do small tasks to earn money and then donate part of their earnings.

## 1 Objective

The data was obtained to study individual donations toward international conservation and how these donations could change under different signals regarding how much others are giving. We focused on the conservation of the habitats of the Montagu's Harrier, an endangered species that migrates from Denmark to Spain to Ghana for wintering and back to Denmark for breeding. The data allows the reproduction of all the statistical analysis and results of the original article, and hence, contributes to a more open science. The data article adds value to the published article [1] and related studies on support for conservation within and efforts across borders, by making a unique data set available for comparative and deeper exploratory studies.

## 2 Data Description

### 2.1 Downloading the Data

A zip file containing a ReadMe document, a Codebook in text format, 4 Stata do-files, a dataset in DTA format, the same dataset in CSV format, and 12 supplementary material files in PDF can be downloaded from Mendeley Data. The total size of the data file in DTA and CSV formats are 64KB and 148 KB, respectively.

### 2.2 Reading the Data

The data was created and analyzed using Stata/MP 13.0 for Mac (64-bit Intel) and is compatible with any Stata/MP or Stata/SE versions 13.0 and higher for both Mac and Windows. The data can also be read in R with the “haven” package and in Python with Pandas. To use the data, unzip the downloaded zip file and import the file named “data.dta” in either Stata or R. The data is labelled in Stata, but a codebook is provided for non-Stata users. To allow data use beyond Stata, R, and Python, we have also included a CSV version of the dataset.

To run all the do-files in Stata, open “00 DIR_Mac.do” for Mac users and “00_DIR_Win.do” for Windows users, change the directory of the main folder (i.e., Line 57) to point to where your unzipped folder is located. For Mac users, you can then run the entire do-file. This should automatically install all needed programs and run all analysis conducted for the original article. For Windows users, save and close the file after changing the directory. When you run the do-files for the analysis conducted in the original article (i.e., “01 Analysis.do” and “02 Graphs.do”), first run the command “do 00_DIR_Win.do” (i.e., Line 18) before running the rest of the do-file.

### 2.3 Structure of the Data

The data is in long format. Each column is a variable, and each row is a participant. The data contains 38 variables and 716 observations. For anonymity, each participant is given a subject ID number (i.e., “subjectid”). Subjects are also identified based on where they had the experiment (i.e., “countryid==1” if Denmark, “countryid==2” if Spain, and “countryid==3” if Ghana), the experiment treatment they were in (i.e., “maintid” and “treatid”) which corresponds to the session they were in (i.e., “sessionid”). We provide more details regarding the treatment and the sessions below. Table 1 provides a description of all variables in the dataset. For variables related to the exit questionnaire, the actual questions are available as a supplementary material uploaded in Mendeley Data.

**Table 1:** Description of variables in the dataset.

Variable Name	Storage Type	Display Format	Variable Label
subjectid	byte	%10.0g	Subject ID Number In Treatment
smalleff	int	%10.0g	Answer to Small Effort Game
mistakes	byte	%10.0g	Deviation from Correct Answer in Small Effort Game
dictokens	byte	%10.0g	Token Endowment for Dictator Game
donated	byte	%10.0g	Amount Donated by Participant
kept	byte	%10.0g	Amount Kept by Participant
session	byte	%10.0g	Session ID
countryid	long	%8.0g	Country ID
maintid	long	%8.0g	Treatment ID
treatid	long	%8.0g	Treatment ID + Country
signal	float	%9.0g	Signal Given to Participants
percentgive	float	%9.0g	Percentage Given
percentkept	float	%9.0g	Percentage Kept
age	byte	%10.0g	Age of Participant
gender	byte	%10.0g	Gender of Participant
believe	byte	%10.0g	Believe that Others Exist
don_locchar	byte	%8.0g	Previously Donated to Local Community
don_church	byte	%8.0g	Previously Donated to Church
don_genchar	byte	%8.0g	Previously Donated to Humanitarian
don_locenvi	byte	%8.0g	Previously Donated to Envi Project
don_natenvi	byte	%8.0g	Previously Donated to National Envi Org
don_intenvi	byte	%8.0g	Previously Donated to International Envi Org
spent_money	double	%10.0g	Money Donated Last Year
spent_currency	str3	%9s	Currency of Money Donated Last Year
spent_time	double	%10.0g	Time Donated Last Year
look_birds	byte	%17.0g	Enjoy Looking at Birds
conserve_envi	byte	%17.0g	Conserve the Environment
mbirds	byte	%17.0g	Look at Bird in Nature
birdlife	byte	%17.0g	Familiar with BirdLife International
dic_all	byte	%17.0g	Conservation will happen in all countries
dic_iff	byte	%17.0g	Conservation will happen only if all countries
dic_but1	byte	%17.0g	Conservation will not happen if at least one country
rank_natgov	byte	%10.0g	Rank – Trust in National Government, 1 highest
rank_pubinst	byte	%10.0g	Rank – Trust in Public Institution, 1 highest
rank_local	byte	%10.0g	Rank – Trust in Local City County, 1 highest
rank_int	byte	%10.0g	Rank – Trust in International NGO, 1 highest
rank_people	byte	%10.0g	Rank – Trust in People, 1 highest
rank_business	byte	%10.0g	Rank – Trust in Private Company, 1 highest

Table 2 provides summary statistics (i.e., number of observations, mean, standard deviation, minimum value, and maximum value) for each of the variables the dataset. The variable “spent_currency” is a string and contains 237 observations with “DKK”, 237 observations with “EUR’ and 248 observations with “GHS”. DKK, EUR, and GHS stand for Danish krone, euro, and Ghanian cedi, respectively. All countries had 20 sessions, and all sessions had 12 participants each, except for Session 13 in Denmark (which only had 10 participants) and Session 15 in Denmark and Spain (which only had 11 participants each). Hence, we have 716 participants across three countries.

**Table 2:** Summary statistics of variables in the dataset.

Variable Name	Obs	Mean	Std. Dev.	Min	Max
subjectid	716	6.49	3.45	1	12
smalleff	716	185.60	7.88	86	264
mistakes	716	2.02	7.62	0	100
dictokens	716	19.66	1.47	0	20
donated	716	4.95	4.85	0	20
kept	716	14.72	4.98	0	20
session	716	10.48	5.78	1	20
countryid	716	2.00	0.82	1	3
maintid	716	2.85	1.20	1	4
treatid	716	3.30	1.74	1	6
signal	322	43.08	18.76	15	77.5
percentgive	716	25.22	24.65	0	100
percentkept	716	74.64	24.79	0	100
age	713	22.50	3.64	2	56
gender	712	0.52	0.50	0	1
believe	709	0.81	0.39	0	1
don_locchar	714	0.43	0.50	0	1
don_church	714	0.23	0.42	0	1
don_genchar	714	0.36	0.48	0	1
don_locenvi	714	0.12	0.33	0	1
don_natenvi	714	0.08	0.26	0	1
don_intenvi	714	0.13	0.34	0	1
spent_money	710	270.34	747.36	0	10000
spent_curr∼y	N/A	N/A	N/A	N/A	N/A
spent_time	687	57.32	179.45	0	1512
look_birds	712	3.22	0.72	0	4
conserve_e∼i	711	2.90	1.00	0	4
mbirds	711	2.00	1.13	0	4
birdlife	709	1.31	0.90	0	4
dic_all	708	2.44	1.22	0	4
dic_iff	705	2.70	1.24	0	4
dic_but1	703	1.92	1.18	0	4
rank_natgov	703	4.32	1.75	0	9
rank_pubinst	704	2.84	1.30	1	7
rank_local	702	3.88	2.39	0	54
rank_int	706	2.36	1.46	1	9
rank_people	701	2.53	1.57	1	10
rank_busin∼s	703	3.88	1.89	1	10

## 3 Experimental Design, Materials and Methods

### 3.1 Experiment Design

**Fig. 1:**
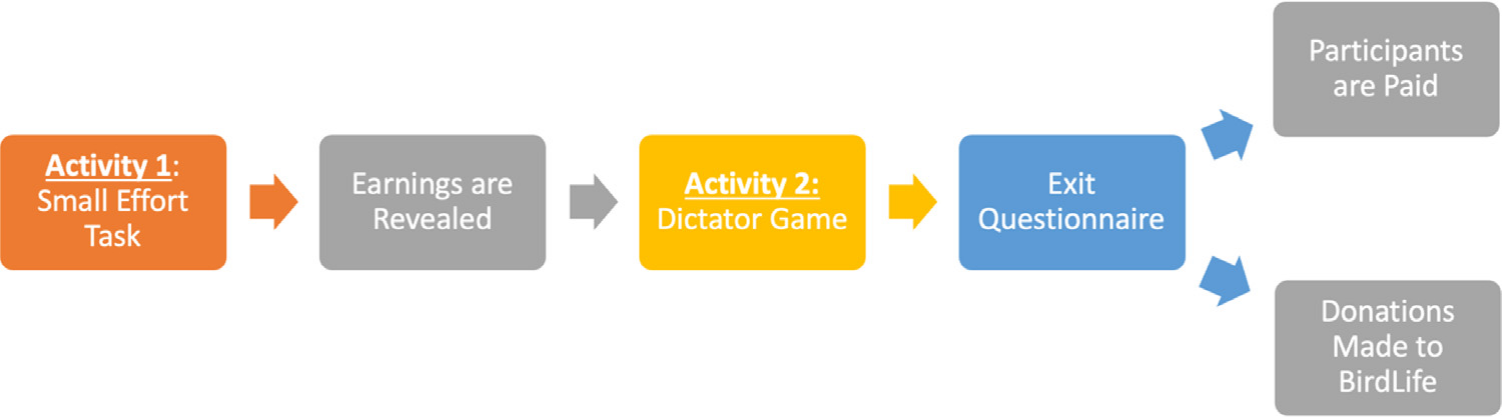
Experimental design overview.

Fig. 1 provides an overview of our experimental design. Participants engage in two activities: a small effort task and a dictator game. In the small effort task, individuals are told to count the number of 1’s in a page filled with 1’s and 0’s. For instance, in the series “00000111010101010101”, there are 9 1’s. For each deviation from the correct answer, a participant loses 1 E$ (experimenter dollar) from the 20 E$ they were initially given. All participants had 5 minutes to complete this activity. There were 186 1’s (see the file “SM – 02 SmallEffort S” uploaded in Mendeley Data [2]).

Participants were informed of their earnings after Activity 1. They were then asked how much of their earnings they would like to donate to conserve the habitats of the Montagu's Harrier (i.e., Activity 2). Participants were informed that the amounts donated were summed up and donated to BirdLife International, an organization known to engage in conservation work related to migratory bird species like the Montagu's Harrier. Apart from this, a photo of the Montagu's Harrier and its habitat, and the information given at each treatment, participants were not given other information regarding the bird or its habitat (i.e., no information was given regarding its rate of decline or the urgency to help it). All participants received a certificate of donation. A copy of this certificate is available as a supplementary material uploaded in Mendeley Data.

In Activity 2, participants were given different information depending on which treatment the session to which they were randomly assigned. Table 3 describes each of our Activity 2 treatments. Each participant is allowed to only join one session and is only assigned one treatment. An English version of all experiment instructions is available as a supplementary material uploaded in Mendeley Data.

**Table 3:** Treatment Name and Description.

Treatment Name	Treatment Description
T0: Baseline Information	Participants were asked to donate
T1: Collaboration Information	T0 + Participants were informed that the bird migrates and that participants in the other two countries were also being asked to donate
T2: Forwarding Information	T1 + Participants are told that their donation amounts will be shared with other participants
T3D: Donation Information Denmark	T1 + Participants are given information about how much individuals in Denmark donated
T3S: Donation Information Spain	T1 + Participants are given information about how much individuals in Spain donated
T3G: Donation Information Ghana	T1 + Participants are given information about how much individuals in Ghana donated

After Activity 2, participants are asked to answer a short exit questionnaire which collected their socio-demographic information and their environmental and donation preferences. In the exit questionnaire, participants were asked if they have donated time or money in the last year (and how much, if they have), and their level of agreement towards sentences like “I conserve the environment” and “I look at birds in nature”. While the participants answered the exit questionnaire, the experimenter prepared both the participant payment and the donation to BirdLife International. At the end of each session, participants knew that a donation to BirdLife International had been made.

### 3.2 Pilot Testing

This experiment is one of several experiments conducted at the same time, using the same infrastructure (see Abatayo and Thorsen [3], Bull, et al. [4], and Abatayo, et al. [5]). To ensure all experiments proceeded as planned and risks are properly managed, a pilot study with all the instructors and experimenters for all experiments was conducted in Denmark with Danish, Spanish, and Ghanaian participants. No data was collected during the pilot. The pilot tested the logistical infrastructure in place and was used to time each experiment. Back-up plans were created in case of logistical failure (e.g., no internet or electricity in Ghana).

### 3.3 Experimenter Training

All sessions in all countries had an instructor and an experimenter, and all instructors and experimenters were trained in Denmark prior to the experiment. The instructor interacted with the experiment participants. They gave instructions, answered questions, and collected participant decision sheets for Activities 1 and 2. Instructors were locals of the country that the experiment was being conducted, and they were able to speak the local language and dialect (i.e., in Spain, the instructor was able to speak both Catalan and Spanish since the experiments were conducted in Barcelona). In contrast, the experimenter only interacted with the participants at the end of the experiment when paying the participants. Experimenters received the participant decision sheets from the instructor and inputted the decisions in a Google Sheet shared by all experimenters in all three countries.

### 3.4 Experiment Materials

**Fig. 2:**
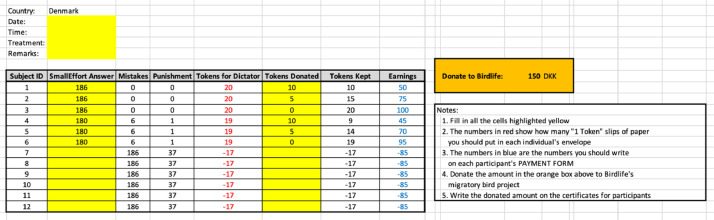
Sample of the Google sheet file shared across experimenters.

Fig. 2 provides a sample of the Google Sheet file that is shared across experimenters in Denmark, Spain, and Ghana. Each session is a separate file which was then appended across session and merged with answers from the exist questionnaire. The experimenter fills in details for everything highlighted in yellow. Participant answers to Activity 1 is in the second column. The file automatically computes the deviation from the correct answer and the corresponding punishment because of the deviation. In Activity 2, participants start off with the E$ displayed in the column “Tokens for Dictator”.

Other experiment materials, such as the donation certificate and the photo of the habitats of the Montagu's Harrier, can be found as a supplementary material uploaded in Mendeley Data.

### 3.5 Recruitment of Participants

Experiments were conducted at the University of Copenhagen (Denmark), Pompeu Fabra University (Spain), and University of Ghana (Ghana), and participants were university students. In Denmark and Spain, participants were recruited using the Online Recruitment System for Economic Experiments (ORSEE) [6]. ORSEE contains a database of participants from which 100 participants are randomly selected and invited to a session. In each session, 36 slots are available for sign-up on a first come first serve basis. Once the slots are filled, participants who have not signed up are “put back” in the database, in which they will have a chance to be one of 100 that will be selected for another session. A similar system, albeit manual, was implemented in Ghana. Students were recruited using flyers and in-class advertisements and then randomly invited to sessions.

### 3.6 Experiment Protocol

Experiments were conducted in accordance to standard experimental economics protocols. Participants in Denmark and Spain were individually seated in tables with partitions, while participants in Ghana were seated in two seats apart. Before the start of the experiment, participants were asked to put their belongings aside and switch their mobile phones to silent mode. Participants were only allowed to communicate with the Instructor and they had to wait to be called by the Instructor before speaking. All participants signed a consent form. No personal identifying information of the participants were kept. The experiments were conducted in April 2014.

## Ethics Statements

Our research involved human subjects. In accordance with the Danish legislation, we obtained a clearance under the Danish Protection Act (REF: 2015-15-0117) to process the data obtained from our experiments. We confirm that the relevant informed consent was obtained from our subjects and that participants were advised that they were free to leave at any time during the experiment.

## CRediT authorship contribution statement

**Anna Lou Abatayo:** Conceptualization, Methodology, Software, Validation, Formal analysis, Investigation, Resources, Data curation, Writing – original draft, Writing – review & editing, Visualization, Project administration. **Mathias Vogdrup-Schmidt:** Conceptualization, Methodology, Investigation. **Jason F. Shogren:** Conceptualization, Methodology, Validation, Writing – review & editing. **Niels Strange:** Conceptualization, Methodology, Validation, Resources, Writing – review & editing, Supervision, Funding acquisition. **Bo Jellesmark Thorsen:** Conceptualization, Methodology, Validation, Resources, Writing – review & editing, Supervision, Funding acquisition.

## Declaration of Competing Interest

The authors declare that they have no known competing financial interests or personal relationships that could have appeared to influence the work reported in this paper.

## Data Availability

Dataset for "Factors Affecting Support for Transnational Conservation of Migratory Species" (Original data) (Mendeley Data).
